# Late Holocene Paleodietary Patterns Among the Ancestral Ohlone: Ecogeographic Partitioning of Resources Along the San Francisco Bay Eastern Shore

**DOI:** 10.1002/ajpa.70112

**Published:** 2025-08-20

**Authors:** Melanie M. Beasley, Eric J. Bartelink, Alan Leventhal, Monica V. Arellano, Richard Massiatt, Charlene Nijmeh

**Affiliations:** ^1^ Department of Anthropology Purdue University West Lafayette Indiana USA; ^2^ Anthropology Department California State University, Chico Chico California USA; ^3^ Department of Anthropology Muwekma Ohlone Tribal Archaeologist and Ethnohistorian, San Jose State University San Jose California USA; ^4^ Former Tribal Vice Chairwoman and Most Likely Descendant, Muwekma Ohlone Tribe of the San Francisco Bay Area Manteca California USA; ^5^ Tribal Councilman, Executive Director of Cultural Resources and Most Likely Descendant, Muwekma Ohlone Tribe of the San Francisco Bay Area Manteca California USA; ^6^ Chairwoman, Muwekma Ohlone Tribe of the San Francisco Bay Area Manteca California USA

**Keywords:** fisher‐hunter‐gatherers, Muwekma Ohlone, paleodiet, stable carbon isotopes, stable nitrogen isotopes

## Abstract

**Objectives:**

In the San Francisco Bay Area, Late Holocene resource intensification models predict an increase in diet breadth and a reduction in foraging efficiency associated with an increase in population, sedentism, and territoriality among fisher‐hunter‐gatherer populations. Here we compare bone stable carbon (*δ* ^13^C) and nitrogen (*δ* ^15^N) isotope values of sites along the eastern bay shore to interpret how ecogeographical partitioning of resources by latitude and distance from the bay reflects differential access and control of resources.

**Materials and Methods:**

With the support of, and in collaboration with, the Muwekma Ohlone Tribe of the San Francisco Bay Area, we analyzed bone collagen and bioapatite from 154 burials from *Mánni Muwékma Kúksú Hóowok Yatiš Túnnešte‐tka* (CA‐ALA‐329) (2500–180 cal B.P.) to examine temporal changes in diet. Further, we examined the ecogeographical partitioning of resources in relation to published data from four contemporaneous sites.

**Results:**

For collagen (*n* = 146), *δ* ^13^C values average −18.2‰±0.7‰ (1SD) and *δ* ^15^N values average 9.8‰±1.5‰ (1SD). For bioapatite (*n* = 144), *δ* ^13^C values average −13.8‰±1.0‰ (1SD). Adult males had statistically significantly higher isotope values compared to adult females, but differences were minimal (< 1.1‰). No meaningful temporal changes in diet were identified. Regional dietary differences occurred along a latitudinal gradient of the eastern bay shore.

**Discussion:**

Foraging efficiency was localized, suggesting that the exploitation of different microhabitats was critical to each tribal group. Stable isotope data complement zooarchaeological and paleobotanical data; although they reveal different aspects of subsistence practices and diet.

## 1 Introduction

Pre‐contact California had one of the highest estimated population densities in North America based on the number of radiocarbon‐dated site components, sites, and burials (Baumhoff 1963; Ubelaker 2006). In the San Francisco Bay Area of Central California, archeologists documented over 425 shell mound sites that may have functioned as village sites, ceremonial centers, and/or burial mounds (Moratto 1984; Milliken et al. 2007; Nelson 1909; Leventhal 1993). Archaeological evidence of population growth and increased sedentism through time is associated with an expansion in diet breadth, marked by an increased reliance on wild plant resources, such as acorns and small seeds (Basgall 1987; Wohlgemuth 2004, 2016) and a decline in the relative abundance of high‐ranked, large vertebrate fauna relative to lower‐ranked, smaller vertebrate fauna identified in archaeological assemblages (Broughton 1994, 1997, 1999, 2002; Broughton et al. 2007, 2013, 2015; Simons 1992). These trends among fisher‐hunter‐gatherer peoples are interpreted within the framework of archaeological resource intensification models, which predict a decline in foraging efficiency during the Late Holocene in Central California. In densely populated regions, such as the eastern shore of the San Francisco Bay, the ecogeographical partitioning of resource patches would have provided access to, and control of, critical food resources by different groups based on factors such as latitude and distance from the bay shore.

With the support of, and in collaboration with, the Muwekma Ohlone Tribe of the San Francisco Bay Area, this study uses stable carbon and nitrogen isotope analysis of human burials from sites along the eastern shore of San Francisco Bay to evaluate the relationship between ecogeographical partitioning and food consumption patterns during the Late Holocene. We first report new data for adults and juveniles from *Mánni Muwékma Kúksú Hóowok Yatiš Túnnešte‐tka*, the “Place Where the People of the Kúksú Pendants are Buried Site” (CA‐ALA‐329, also known as the Ryan Mound). In addition to dietary reconstruction among individuals interred at the site and a comparison of temporal site components, a second aim of this study is to contextualize the site within the broader eastern bay shore through comparison with previous stable isotope studies (Bartelink 2009; Beasley et al. 2013; Gardner 2013). Thus, by comparing isotope data from the site to four other eastern bay shore sites (CA‐CCO‐295, CA‐ALA‐309, CA‐ALA‐328, and *Yukisma* Mound, “At the Oaks” CA‐SCL‐38), we explore how dietary patterns relate to ecogeographic partitioning of key dietary resources.

## 2 Background

### 2.1 Resource Intensification Models in the San Francisco Bay Area

Archeologists working in the Bay Area have long been interested in human impacts on the Indigenous landscape and local food resources, especially considering the region's high population density. Late Holocene Central California was marked by incremental population growth, a high degree of sedentism, and territorial circumscription of cultural boundaries (Basgall 1987; Beaton 1991; Jones and Klar 2007; Rosenthal et al. 2007). Several studies have documented a temporal decline in the relative abundance of large game relative to smaller game through zooarchaeological analyses of faunal assemblages from several Bay Area mounds (Broughton 1994, 1997, 1999, 2002; Broughton et al. 2007, 2013, 2015; Simons 1992). In addition, researchers have documented the increased use of acorns (from various oak species, *Quercus* spp. and *Lithocarpus* sp.) as well as the mortars and pestles used to process them at several Bay Area sites (Basgall 1987; Broughton 1999; Wohlgemuth 2004, 2016). Resource intensification models, derived from foraging theory, help explain temporal patterns in resource use during the Late Holocene. Based on the relationship between prey body size and return rate in kilocalories (i.e., the prey‐rank model), human foragers should optimize caloric returns by acquiring the largest game (high‐ranked) resources regardless of their abundance on the landscape and only resort to smaller prey and wild plants (i.e., low‐ranked resources) when high‐ranked resources are substantially depleted on the landscape (Charnov et al. 1976; Stephens and Krebs 1986).

Using prey‐rank as a proxy for human foraging behavior, resource intensification models argue that the growing human population within the Bay Area exerted predation pressure on local high‐ranked large game resources, such as elk, deer, large marine mammals, and sturgeon, resulting in a decline in their relative abundance through time (Broughton 1994, 1997, 1999, 2002; Broughton et al. 2007, 2013, 2015). In turn, human foragers shifted their emphasis toward more costly to acquire and/or process (i.e., lower‐ranked) resources, such as sea otters, shellfish, small terrestrial mammals, and vegetal resources (e.g., acorns, small seeds, and geophytes). Probable evidence of declining foraging efficiency has been identified in Bay Area site components using stable isotope data of human burials as well as various archaeofaunal, paleobotanical, and artifact indices that track relative abundance through time (Bartelink 2009; Beasley et al. 2013; Broughton et al. 2023). Notably, declines in foraging efficiency were noted in sites from the *Mayyan Šaatošikma* (translated as the Coyote Hills Sites, including *Muwékma Kúksú Hóowok Yatiš Túnnešte‐tka*) by Broughton (1994), who found a significant temporal decline in the relative abundance of artiodactyls relative to sea otters. Recently, Broughton et al. (2023:1) evaluated resource depression and distant patch use of tule elk by comparing a Bay Area site and Central Valley site underscoring that “human behavioral responses to resource depression can vary in relation to local ecology.” The Central Valley data suggested that local depression spurred the use of distant elk patches; in contrast, the Bay Area data indicated a limited local elk habitat that did not support the use of distant elk patches, suggesting a widening of diet breadth as found by previous isotope studies in the region (Bartelink 2009; Broughton et al. 2023).

### 2.2 Environmental Context

**FIGURE 1:**
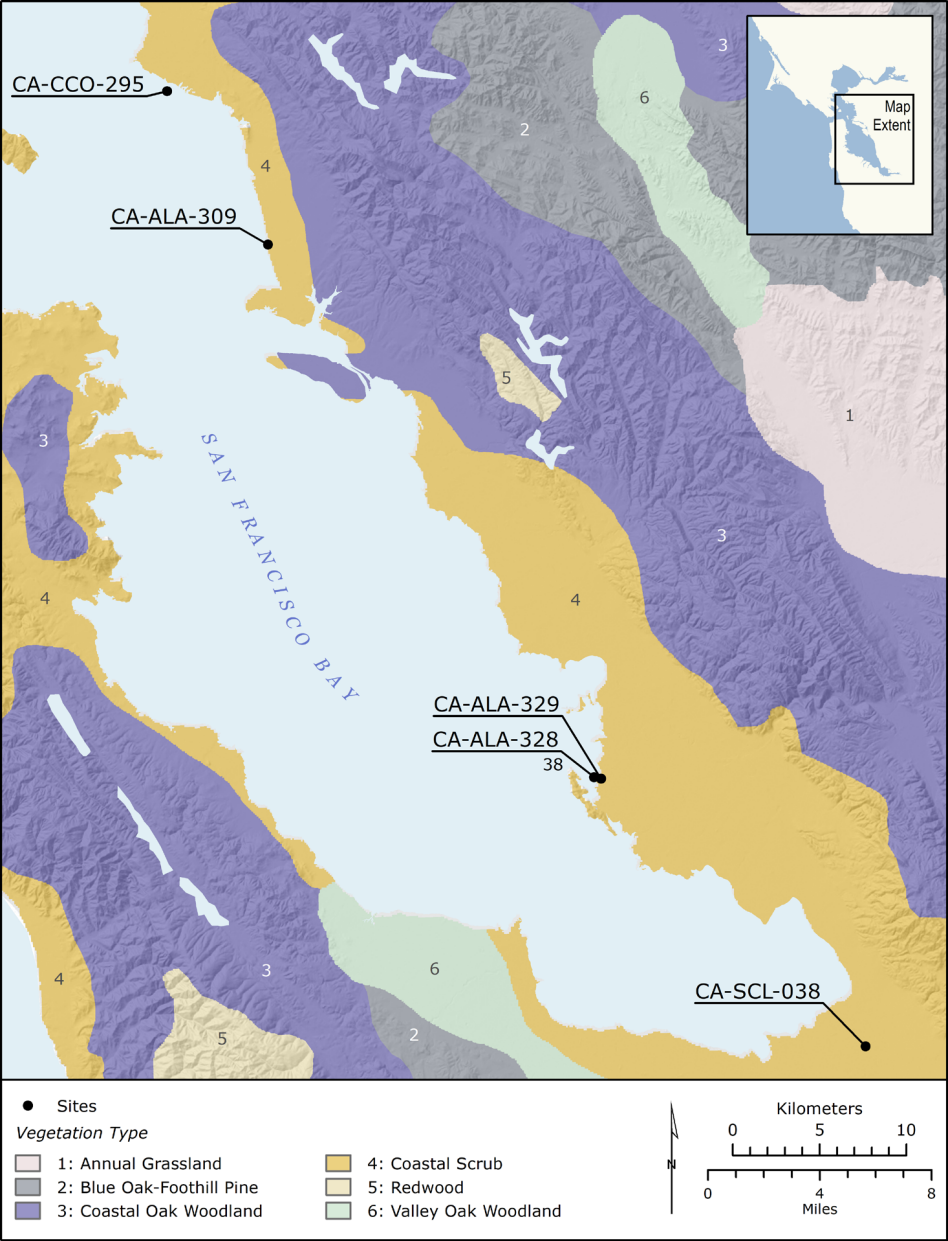
Map of archaeological sites discussed in this study with reference to local vegetation habitat adapted from Whitaker et al. (2020: Figure 192, pp. 466).

The San Francisco Bay estuary encompasses a multitude of habitats, which provide a variety of niches for flora and fauna (Figure 1). Terrestrial and marine resource productivity varies between different regions of the Bay Area, with the northern reaches (open bay) most closely approximating a coastal environment abundant in high trophic level marine resources; and the southern reaches defined by marshland and mudflats abundant in terrestrial plant and animal resources and low trophic level aquatic invertebrates (Arpaia and Wohlgemuth 2016; Broughton 1994; Schoenherr 1992; Whitaker and Byrd 2014).

*Mánni Muwékma Kúksú Hóowok Yatiš Túnne šte‐tka* is located along the southeastern bay shore within a saltmarsh. The site is within a coastal scrub zone and is situated near four distinct wetland environments, including open water, tidal salt marshes, freshwater marshes, mudflats, as well as grasslands and oak woodlands, which would have provided access to a wide range of terrestrial and aquatic resources (Schoenherr 1992). The Ohlone navigated the open waters of the San Francisco Bay and smaller waterways using tule balsas (boats), which provided access to marine, estuarine, and freshwater fish, shellfish, waterfowl, crab, and marine mammals (e.g., sea otter, sea lion, harbor seal, fur seal). Tidal salt marshes and freshwater marshes provided access to numerous species of birds as well as bird eggs, salt, and plant materials used for building boats, basketry, mats, and housing (Hylkema 2002). Terrestrial plant communities include valley oak savannah, California prairie, and blue oak‐California foothill pine forest, which were home to mammals such as artiodactyls (black‐tailed deer, elk, pronghorn), leporids (jackrabbit and cottontail rabbit), bear, raccoon, coyote, mountain lion, and bobcat (Broughton 1999; Küchler 1977; McBride 1974). Additionally, these environments provided key vegetal resources such as acorns, geophytes, and perennial grass seeds.

*Mánni Muwékma Kúksú Hóowok Yatiš Túnnešte‐tka* lies near CA‐ALA‐12, CA‐ALA‐13, and CA‐ALA‐328, which collectively form a cluster of mounds located within a 1.6 km area. These sites are near the Coyote Hills and share a common environmental context. Although now located a few kilometers from the bay shore, at the time of their use, these mounds were on the shoreline and would have been inundated periodically by seasonal flooding (Bickel 1981; Coberly 1973; Leventhal 1993; Wilson 1999). The major source of freshwater is Alameda Creek located 4.8 km away; although springs were noted historically in the area (Bickel 1981).

To the north along the same shoreline are the contemporaneous mound sites, the Emeryville Shellmound (CA‐ALA‐309) and the Ellis Landing site (CA‐CCO‐295), located in the modern cities of Emeryville and Richmond, respectively. These sites are adjacent to open bay and California prairie, providing access to high trophic level marine resources such as estuarine fish, anadromous fish, crabs, waterfowl, and marine mammals; low trophic level resources such as shellfish; and terrestrial game such as artiodactyls and leporids (Broughton 1994). To the south of *Mánni Muwékma Kúksú Hóowok Yatiš Túnnešte‐tka* in the Santa Clara Valley is the *Yukisma* Mound (CA‐SCL‐38), located in the modern city of Milpitas. *Yukisma* Mound is in a coastal prairie‐scrub mosaic environment located to the south of the tidal marshlands (Whitaker and Byrd 2014). The prairie‐scrub habitat is home to artiodactyls such as elk and pronghorn (Hylkema 2002). The adjacent tidal marshlands include networks of sloughs and tidal mudflats, where shorebirds, waterfowl, horn snails, shellfish, and estuarine and freshwater fish are abundant (Hylkema 2002). Further to the south and the east are oak woodlands, which are prime deer habitats.

### 2.3 Culture History

At Spanish contact in 1769, the San Francisco Bay Area was populated by a diverse number of Native California groups, representing a myriad of languages, including Southern Pomo, Patwin, Bay Miwok, Coast Miwok, Wappo, Karkin Ohlone (Northern Costanoan), and San Francisco Bay Ohlone (Costanoan) (Milliken et al. 2007:99; Milliken et al. 2009:6, 33). The current study area includes the eastern bay shore and a portion of the southern bay shore of the San Francisco Bay, representing the San Francisco Ohlone language group. The region was partitioned into a series of small Ohlone tribal groups with distinct geographic boundaries. These tribal groups consisted of small polities of autonomous, self‐governing groups of around 200–400 individuals who were under the leadership of a headman (Kroeber 1925; Milliken 1995). Population estimates for the Bay Area in the late Holocene ranged from about 3–6 persons per square mile (Milliken 1995; Milliken et al. 2007:99).

### 2.4 Site Context

*Mánni Muwékma Kúksú Hóowok Yatiš Túnnešte‐tka* (CA‐ALA‐329) is a large, ovate earthen mound site, measuring 91 by 122 m at its base, with a height of approximately 3 m from the surface (Wilson 1993). The site is approximately 3.2 km from the eastern shoreline of San Francisco Bay and south of Alameda Creek and Coyote Slough, near the modern cities of Newark and Union City (Bickel 1981). A portion of the site was disturbed in the early 20th century due to the construction of a house and reservoir by landowners (Coberly 1973), which removed the top layer of the mound (Leventhal 1993). The mound was excavated several times, including by W.R. Wedel (1935) and C.E. Smith of U.C. Berkeley (1948), B. Gerow of Stanford University (1959–1968), J. Hester and D. Pritchard of San Jose State College (1962–1968; now San Jose State University), and C.E. Smith of Hayward State University (1972; now California State University East Bay) (Leventhal 1993; Wilson 1999). These excavations resulted in the disinterment of nearly 500 individuals and 48,670 artifacts made of shell, bone, and stone, 91% of which were associated with burials (Leventhal 1993). In 1992, Stanford University reinterred 144 of these individuals just before the full implementation of NAGPRA (Jurmain et al. 2009). Thus, with the support and collaboration of the Muwekma Ohlone Tribe, the current study focuses on individuals from the San Jose State University and UC Berkeley excavations.

Ancestral Ohlone families constructed the mound as a ceremonial structure and place to inter and honor their ancestors (Leventhal 1993). Radiocarbon dates, obsidian hydration dates, and time‐sensitive artifacts indicate that the Ohlone used the mound to bury their ancestors between 2500 and 180 years cal B.P. Leventhal's (1993) detailed analysis of excavation records and artifact assemblages identified 10 temporal components at the site based on obsidian hydration, shell bead and ornament exchange networks (i.e., Scheme B1; Bennyhoff and Hughes 1987), and radiocarbon dates. Subsequent radiocarbon dates of additional human bone, *Olivella* shell beads, and charcoal samples further refined the temporal placement of burials from the site within the accepted Central California dating schemes (Bennyhoff and Hughes 1987; Buonasera 2013; Fournier et al. 2022; Groza 2002; Hughes and Milliken 2007). Following Fournier et al. (2022), for this study, the burials were placed broadly in two temporal components, the Middle Period (2500 to 1000 cal B.P.) and the Late Period (1000 to 180 cal B.P.). Disturbance of the top layers of the mound may have obliterated protohistoric and historical deposits, suggesting the Ohlone were still using the site when the Spanish arrived in 1769.

The presence of numerous N Series banjo‐shaped abalone pendants found within burials links the site to the ethnographically documented *Kuksu* religious tradition (see Bean and Vane 1978; Kroeber 1929, 1932; Leventhal 1993). The *Kuksu* religion's elaborate ceremonies of singing, dancing, and performance in feathered regalia were essential to inter‐community relations, economic exchange, and solidarity between villages and cultures. We interpret *Mánni Muwékma Kúksú Hóowok Yatiš Túnnešte‐tka* as a mortuary mound and not as the village site because burials represent the main archaeological feature at the site, most utilitarian artifacts were found within burials instead of midden contexts, and evidence for stone tool manufacture is absent (Leventhal 1993). Thus, the site is biased toward mortuary behavior and less toward subsistence activities because no village site associated with the mound has been located. Despite these interpretations of the site, there continues to be debate among California archeologists regarding the various functions of burial mound sites throughout Central California, with some finding evidence for their use as village sites atop midden deposits, as ceremonial centers, or strictly used as mortuary complexes (see discussions in Leventhal 1993; Lightfoot 1997; Lightfoot and Luby 2002, 2012; Lightfoot et al. 2017; Luby et al. 2006; Meighan 1987).

### 2.5 Stable Isotopes for Testing Foraging Models

Stable carbon and nitrogen isotope analysis of bone collagen has been used by archeologists for decades to understand the relative contribution of marine versus terrestrial resources consumed by past human populations (Chisholm et al. 1982, 1983; Keegan and DeNiro 1988; Schoeninger et al. 1983; Walker and DeNiro 1986). Stable carbon isotope values (*δ* ^13^C) reflect the different photosynthetic pathways used by terrestrial plants to maximize their efficiency of carbon fixation, whereas in aquatic systems carbon derives from dissolved bicarbonate, aquatic plants, marine algae, phytoplankton, and freshwater inputs containing organic matter (Fry 2006; O'Leary 1981; O'Leary 1988). *δ* ^13^C values distinguish between C_3_ and C_4_ terrestrial plants, but when marine dietary inputs occur, the *δ* ^13^C values overlap with C_4_ terrestrial plants having more positive values relative to those from C_3_ ecosystems (Schoeninger and DeNiro 1984). Stable nitrogen isotope values (*δ* ^15^N) reflect dietary protein and indicate the trophic position of an organism within a food web as each trophic level exhibits a 3‰–5‰ stepwise increase, with marine ecosystems having higher values due to their longer food chains (Caut et al. 2009; Minagawa and Wada 1984; Schoeninger 1995; Schoeninger and DeNiro 1984). In coastal or estuarine ecosystems, elevated *δ* ^15^N values of terrestrial plants may be due to higher source values in soils due to fixation with ^15^N‐enriched saline soils and sea spray effects (Ambrose 1993; Heaton 1987; Sealy et al. 1987).

The relationship of bone *δ* ^13^C values to diet is complex because collagen does not reflect the whole diet (Ambrose and Norr 1993; Tieszen and Fagre 1993; Froehle et al. 2010). Experimental feeding studies indicate collagen *δ* ^13^C values are primarily influenced by dietary protein but with substantial contributions from other dietary macronutrients (i.e., as much as two‐fifths of the carbon atoms derive from intact amino acids) (Fernandes et al. 2012; Froehle et al. 2010; Kellner and Schoeninger 2008; Warinner and Tuross 2009). In contrast, bone bioapatite *δ* ^13^C correlates strongly with dietary *δ* ^13^C values from the whole diet, including carbohydrates, lipids, and protein not used in tissue synthesis, but it alone does not reflect the protein source (Froehle et al. 2010). Kellner and Schoeninger (2008), later revised in Froehle et al. (2010), developed a model that uses both collagen and bioapatite *δ* ^13^C values to model the protein and energy sources in the diet to make interpretations of the whole diet.

Previous stable isotope paleodietary research in California includes regional studies of the Santa Barbara Channel of southern California (Fauvelle and Somerville 2021; Goldberg 1993; Harrison and Katzenberg 2003; Walker and DeNiro 1986), Monterey Bay (Jones 1996; Newsome et al. 2004), the San Francisco Bay Area (Bartelink 2009; Beasley et al. 2013; Eerkens et al. 2024; Gardner et al. 2018; Greenwald et al. 2022), the Sacramento‐San Joaquin Delta region (Bartelink 2006; Eerkens et al. 2013; Greenwald et al. 2016), and northeastern California (Eerkens and Talcott 2018). While numerous studies using stable isotopes to interpret past diet and mobility have focused on Central California, including the San Francisco Bay Area, here we summarize the findings of the relevant eastern bay shore sites within the region. Generally, *δ* ^13^C and *δ* ^15^N values of ancient Ohlone individuals from shell and earthen mound sites along the northeastern bay shore were more marine dependent compared to southeastern bay shore sites (Bartelink 2009). In addition to the geographic trend of high dependence on marine resources among individuals closer to the mouth of the estuary, a temporal trend exists with a shift in diet between the Early Period foragers (4950 to 2150 cal B.P.) compared to Middle and Late Period (2150 to 200 cal B.P.) foragers (Bartelink 2009). The Early Period is characterized by higher *δ* ^13^C and *δ* ^15^N values consistent with consumption of marine and anadromous (e.g., sturgeon) fish and marine mammals, whereas the Middle and Late Period diet shifted to also emphasize shellfish and terrestrial resources, such as deer and C_3_ plants to a greater extent (Bartelink 2009; Beasley et al. 2013; Eerkens and Bartelink 2020).

Early studies in the Bay Area focused on diet reconstructions of adults, but more recently isotopic studies of breastfeeding, weaning, and juvenile foraging have expanded our understanding of diet across the life course (Eerkens and Bartelink 2013; Eerkens et al. 2011, 2017; Gardner 2013; Gardner et al. 2018; Greenwald 2017). Most of these studies focus on serial sections of dentinal collagen of adults that survived into adulthood to understand the impact of breastfeeding and weaning from the first molar (Eerkens and Bartelink 2013; Eerkens et al. 2011, 2017) and childhood diet and juvenile foraging patterns from the second and third molars (Greenwald et al. 2016, 2022). As expected, following studies in other regions, *δ* ^15^N values show the expected increase in trophic level due to breastfeeding, followed by a drop after the cessation of weaning (Eerkens and Bartelink 2013; Eerkens et al. 2011, 2017). At *Mánni Muwékma Kúksú Hóowok Yatiš Túnnešte‐tka* (CA‐ALA‐329), Fournier et al. (2022) analyzed dentinal collagen sections of 39 individuals and found that those who died in childhood were weaned later than individuals who survived into adulthood, suggesting greater investment by breastfeeding mothers who cared for their sick children.

Within Central California, Bay Area individuals have isotope values that plot along a trendline characterized by consumption of marine foods with varied contributions of terrestrial C_3_ foods, while inland Central Valley individuals have distinct values with little contribution from marine resources (Bartelink 2006). Sites located between the Bay Area and Central Valley in the Delta region reflect a transition between the two dietary patterns, showing greater reliance on freshwater fish, terrestrial game (e.g., artiodactyls), and wild plants (e.g., acorns and small seeds) from brackish environments (Bartelink et al. 2020; Eerkens et al. 2013, 2021). The isotope studies discussed above establish biological and cultural links between the past and present by the Muwekma Ohlone Indian Tribe as they continue their efforts to re‐establish their federal recognition through the U.S. government (Panich et al. 2024).

## 3 Material and Methods

### 3.1 Sample Selection

From the *Mánni Muwékma Kúksú Hóowok Yatiš Túnnešte‐tka* skeletal collection, curated by the Department of Anthropology at San Jose State University (SJSU), we sampled 142 individuals for stable isotope analysis following departmental curation and sampling guidelines and with the permission and collaboration of the Muwekma Ohlone Tribe of the San Francisco Bay Area. In addition, we use data from 12 individuals published in Bartelink (2006, 2009) that were sampled from the Phoebe A. Hearst Museum of Anthropology at UC Berkeley. Of the 154 total samples, ribs were selected for 153 individuals, with one individual only having a long bone fragment available for sampling.

Leventhal (1993) provided the burial information (e.g., sex, age, mortuary context, burial‐associated artifacts) used for this analysis. All burials had been previously analyzed following standard scoring methods to estimate sex and age or using methods covered therein (Buikstra and Ubelaker 1994). The temporal period for each burial was assigned by direct radiocarbon dates, estimated from the relative stratigraphic position within the site based on Central California dating schemes, or based on time‐sensitive grave goods (Leventhal 1993; Groza 2002; Hughes and Milliken 2007; Bennyhoff and Hughes 1987). Tables 1 and 2 provide a breakdown of the sample by time period and sex.

**TABLE 1:** Descriptive statistics for isotope and sample quality data in the *Mánni Muwékma Kúksú Hóowok Yatiš Túnnešte‐tka* (CA‐ALA‐329) population with diagenetically altered samples removed from the analysi.

	δ^13^C (col, VPDB, ‰)	δ^15^N (col, AIR, ‰)	Atomic C:N Ratio	Collagen Yield (%)	δ^13^C (bioapat, VPDB, ‰)	Bioapatite Yield (%)
Infant (0–3 years) mean	−18.0	11.6	3.3	15	−14.5	26
SD	0.8	2.5	0.0	6	1.2	4
*N*	9	9	9	9	8	8
Child (3–12 years) mean	−18.9	8.7	3.3	16	−14.1	28
SD	0.9	2.1	0.0	5	0.8	3
*N*	8	8	8	8	8	8
Adolescent (12–18 years) mean	−18.2	9.6	3.3	14	−13.5	28
SD	0.7	1.0	0.0	7	1.0	4
*N*	15	15	15	15	15	15
Adult Female (18+ years) mean	−18.5	9.3	3.3	14	−14.2	30
SD	0.6	1.0	0.0	6	0.8	5
*N*	60	60	60	60	56	50
Adult Male (18+ years) mean	−17.9	10.4	3.3	11	−13.5	30
SD	0.7	1.2	0.0	6	1.0	5
*N*	51	51	51	51	54	49
All Adult (18+ years) mean	−18.2	9.8	3.3	13	−13.8	30
SD	0.7	1.3	0.0	6	1.0	5
*N*	115	115	115	115	114	103
Total mean	−18.2	9.8	3.3	13	−13.8	29
SD	0.7	1.5	0.0	6	1.0	5
*N*	146	146	146	146	144	133

**TABLE 2:** Descriptive statistics for isotope and sample quality data in the *Mánni Muwékma Kúksú Hóowok Yatiš Túnnešte‐tka* (CA‐ALA‐329) population by temporal component for adults (18+ years) with diagenetical altered samples removed from the analysis.

	δ^13^C (col, VPDB, ‰)	δ^15^N (col, AIR, ‰)	Atomic C:N Ratio	Collagen Yield (%)	δ^13^C (bioapat, VPDB, ‰)	Bioapatite Yield (%)
Middle Period mean	−18.2	9.8	3.3	14	−13.7	30
SD	0.8	1.4	0.0	7	1.3	6
*N*	29	29	29	29	31	26
Late Period mean	−18.2	9.8	3.3	12	−13.9	30
SD	0.7	1.2	0.0	6	0.8	5
*N*	86	86	8.6	86	83	77

### 3.2 Stable Isotope Preparation Methods

The macroscopic quality of the human skeletal remains from the site is excellent, with most burials recovered as intact and nearly complete individuals. Rib samples ranging in weight from 2 to 11 g from the SJSU burials were selected and processed following standard protocols for *δ* ^13^C and *δ* ^15^N from bone collagen (Ambrose 1993; Schwarcz and Schoeninger 1991) and *δ* ^13^C from bone bioapatite (Koch et al. 1997). While detailed cleaning and methods for separation into the two bone phases of interest have been previously published (Beasley et al. 2013; Bartelink et al. 2020), the following is a brief description of the protocol. All bone samples were dremeled with a diamond‐studded drill bit to mechanically clean the external surface and then were ultrasonicated in baths of distilled‐deionized H_2_O and 95% and 100% EtOH. For collagen, bone samples were demineralized in a 0.25 M HCl solution over a 2–3 week period, followed by removal of the humic contaminants with a 24 h treatment in 0.125 M NaOH solution and solubilization using a dilute HCl solution. For bioapatite, bone samples were ground into a powder, then sieved through a mesh screen (< 234 μm). Collagen was removed with a treatment in a 1.5% sodium hypochlorite solution for 48 h (replaced once at 24 h) following a 0.04 mL solution/mg sample ratio (Koch et al. 1997). Finally, samples were treated in a 1.0 M solution of dilute acetic acid, buffered with NaOH to a pH of 4.5 in the same solution‐to‐sample ratio for 24 h (replaced once at 12 h) to remove contaminants. All bone preparation methods were completed at the Stable Isotope Preparation Laboratory in the Anthropology Department at California State University, Chico.

Stable isotope analyses for collagen and bioapatite samples were completed at the University of California, Davis in the Stable Isotope Facility and the Department of Earth and Planetary Sciences Stable Isotope Laboratory, respectively. Collagen *δ* ^13^C and *δ* ^15^N were measured by an elemental analyzer (PDZ Europa ANCA‐GSL) peripheral to an isotope ratio mass spectrometer (PDZ Europa 20–20) with an analytical precision of ±0.2‰ for both measures. Specifically, several internal laboratory standards of known isotope composition were used to calibrate the data, including normalization reference standards and quality control measures reported in (supplemental information S1). The bone collagen and bioapatite isotope results accuracy were calculated using the standard uncertainty calculator in Szpak et al. (2017) (see S1). For bone collagen, accuracy (*u*(*bias*)) was estimated to be 0.06‰ for *δ* ^13^C and 0.05‰ for *δ* ^15^N. Bioapatite *δ* ^13^C was measured using a stable isotope ratio mass spectrometer (GVI Optima) with an analytical precision of ±0.04‰ based on calcite standards (NBS‐19 and UCD‐SM92). For bone bioapatite, accuracy (*u*(*bias*)) was estimated to be 0.23‰ for *δ* ^13^C. However, uncertainty for all three measures could not be calculated following Szpak et al. (2017) because replicate measures were not analyzed. The *δ* ^13^C and *δ* ^15^N are reported in permil (‰, parts per thousand) relative to Vienne Pee Dee Belemnite (VPDB) and AIR.

### 3.3 Evaluation of Diagenesis

Sample quality for both fractions of bone was evaluated independently because collagen sample quality is not predictive of bioapatite sample quality (Beasley et al. 2024). To evaluate collagen sample quality, atomic C:N ratios and collagen yields were calculated to ensure biogenic values were obtained from the analysis (Ambrose 1990; DeNiro 1985; van Klinken 1999). All collagen samples were considered to have reliable in vivo values if the collagen yield was above 1% and the atomic C:N ratio was between 2.9 and 3.6. To evaluate bioapatite sample quality, the bioapatite yield was calculated (Chesson et al. 2021). Bioapatite results were considered to have a reliable in vivo value if the bioapatite yield met the acceptable threshold of between 21% and 63% (Chesson et al. 2021). The decision to rely on bioapatite yield as a check for diagenesis was in part a best practice protocol to be minimally destructive of human bone samples and because the method was developed from individuals (*n* = 272) in Central California, including the Yukisma Mound (*n* = 115) included in this study.

## 4 Results

### 4.1 General Results for Mánni Muwékma Kúksú Hóowok Yatiš Túnnešte‐Tka

The stable isotope values and sample quality measures by individual are presented in the (supplemental information S2). A total of eight individuals were either below the acceptable threshold for collagen yield (*n* = 3) or above the acceptable threshold for atomic C:N ratio (*n* = 5). A total of nine individuals were below the acceptable threshold for bioapatite yield, with one individual also having compromised collagen sample quality indicators. Of the 154 individuals included in this study, a total of 146 resulted in reliable in vivo collagen *δ* ^13^C and *δ* ^15^N values, while 145 resulted in reliable in vivo bioapatite *δ* ^13^C values used in further statistical analysis.

**FIGURE 2:**
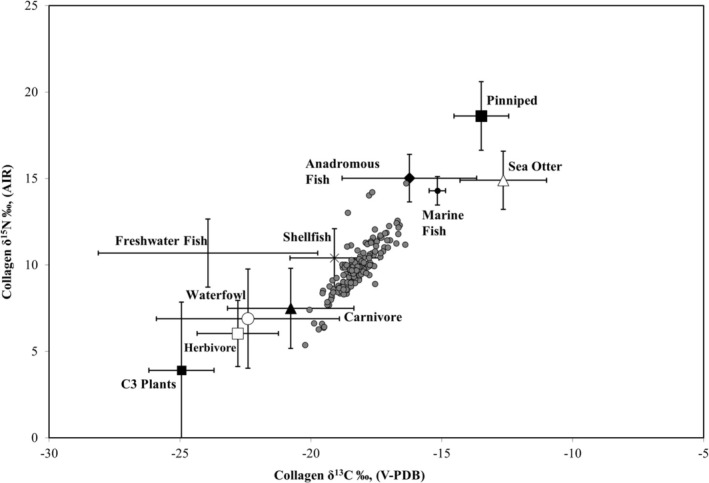
Relationship between the bone collagen *δ* ^13^C and *δ* ^15^N values of individuals from *Mánni Muwékma Kúksú Hóowok Yatiš Túnnešte‐tka* compared to plant and meat food resource values from Central California. The food web data represent sample means and 1 SD error bars of archaeological and modern fauna and modern plants (Bartelink 2006:141, 147–150; Bartelink et al. 2020). Fauna *δ* ^13^C values have been corrected for the diet to tissue offsets (−2.4‰ for herbivores and waterfowl, −3.7‰ for fish) and modern fauna bone corrected for the Suess Effect (1.5‰) to align with archaeological bone values.

**FIGURE 3:**
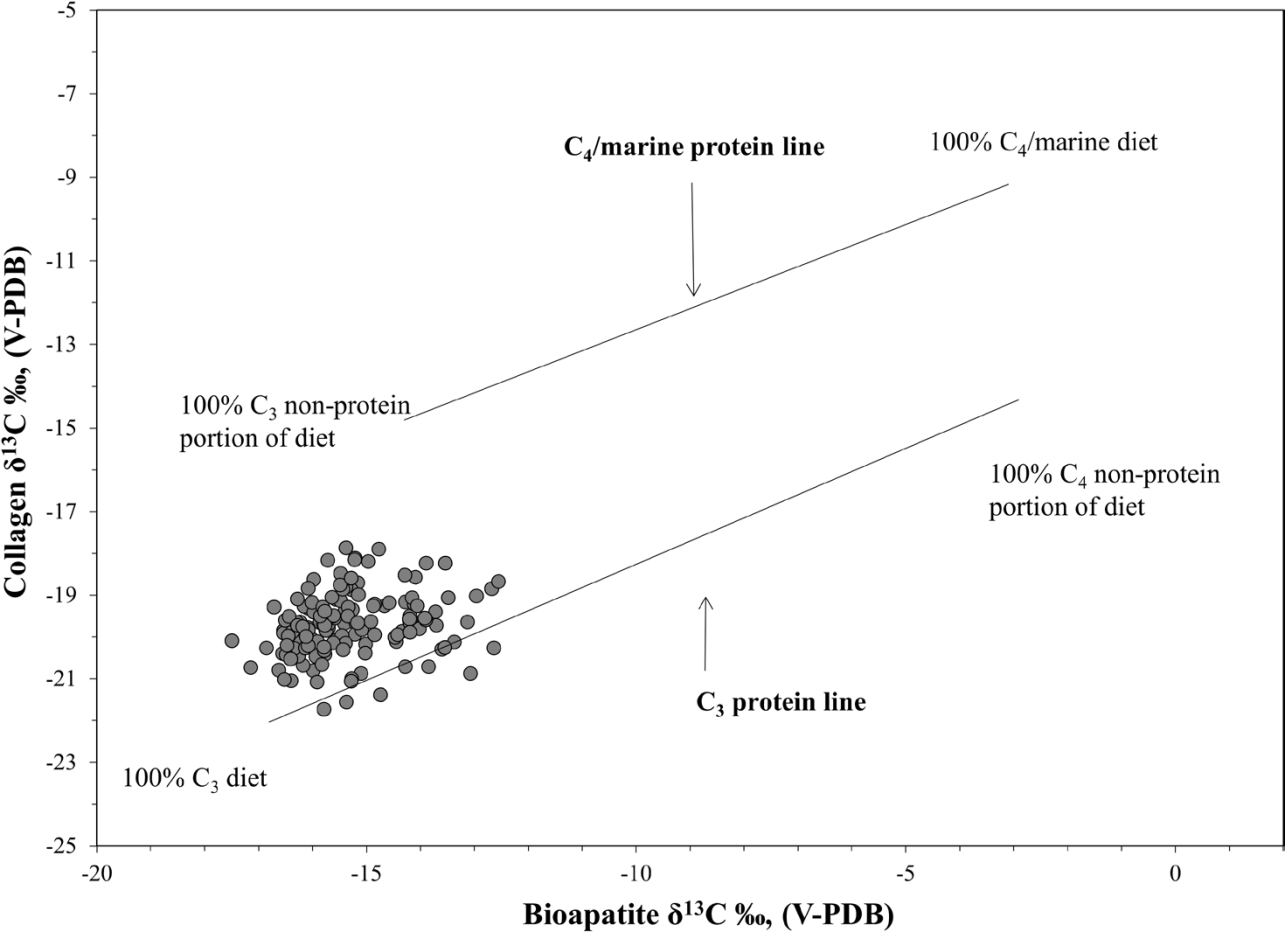
Relationship between bioapatite and collagen *δ* ^13^C values of individuals from *Mánni Muwékma Kúksú Hóowok Yatiš Túnnešte‐tka* compared with the Froehle et al. (2010) model. To account for atmospheric depletion in ^13^C due to fossil fuel burning (i.e., Suess Effect), 1.5‰ was subtracted from each *δ* ^13^C value to align prehistoric individuals with the modern fauna‐based model.

The collagen *δ* ^13^C and *δ* ^15^N and bioapatite *δ* ^13^C descriptive statistics by sex and age category are presented in Table 1. For bone collagen, *δ* ^13^C values vary from −20.2‰ to −16.4‰, with a mean of −18.2‰±0.7‰ (1SD). For bone collagen, *δ* ^15^N values vary from 5.4‰ to 14.7‰, with a mean of 9.8‰±1.5‰ (1SD). In general, the sample falls along the expected trendline for eastern San Francisco Bay shore sites, which indicates consumption of both marine foods and terrestrial C_3_ resources (Figure 2). For bone bioapatite, *δ* ^13^C values vary from −16.0‰ to −11.0‰, with a mean of −13.8‰±1.0‰ (1SD). Figure 3 plots the collagen *δ* ^13^C and bioapatite *δ* ^13^C values to indicate the ratio of C_3_ to C_4_ foods in the diet along the *x*‐axis (bioapatite *δ* ^13^C) and relative position to the protein‐specific regression lines on the *y*‐axis, which discriminate between the C_3_ and marine protein sources (Froehle et al. 2010). The individuals plot closest to the C_3_ protein regression line but clearly reflect dietary inputs from marine protein sources. The non‐protein component of the diet (carbohydrates and lipids) is consistent with the consumption of terrestrial C_3_ resources (Figure 3).

**FIGURE 4:**
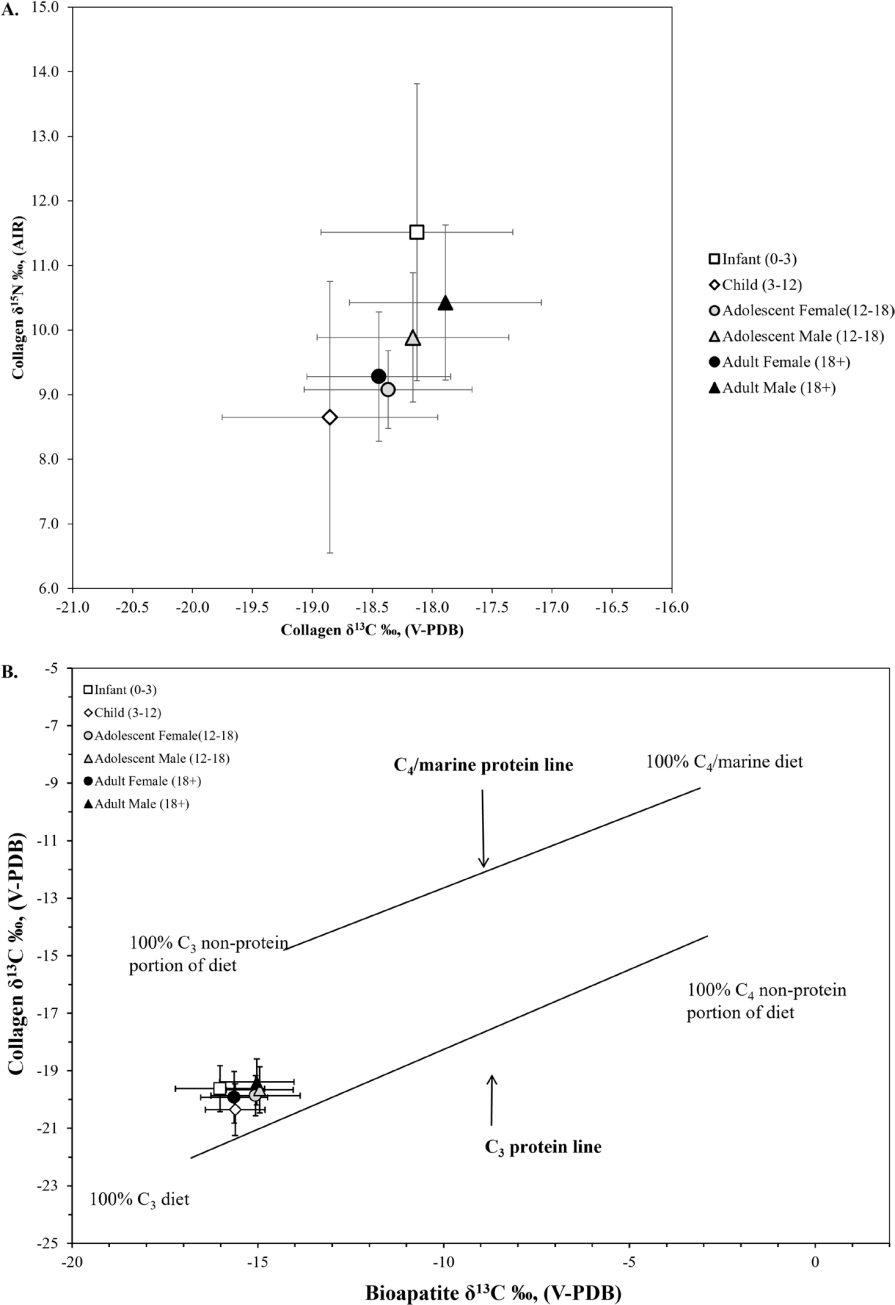
Stable isotope results plotted as sample means with 1 SD error bars for the individuals from *Mánni Muwékma Kúksú Hóowok Yatiš Túnnešte‐tka* by age and sex estimated from the skeletal remains. The protein sources and other dietary macronutrients that impact the bone collagen *δ* ^13^C and *δ* ^15^N values show a trend where infants (0–3 years) have the highest values and children (3–12 years) having the lowest (A). The Froehle et al. (2010) model indicates that when the whole diet is considered, there is no distinct patterning by age and sex, suggesting that food was shared equally among the population (B).

Figure 4 presents the mean values by age and sex. While the focus of our analysis and discussion is on the adults from *Mánni Muwékma Kúksú Hóowok Yatiš Túnnešte‐tka*, it is worth noting that the highest mean values of collagen *δ* ^13^C were for infants (0–3 years; −18.1‰) and the lowest mean values were for children (3–12 years; −18.9‰). Similarly, the *δ* ^15^N values show the same pattern of highest mean values for infants (11.6‰) and the lowest mean values for children (8.7‰). This fits the expected pattern observed in other Central California sites that have high values during breastfeeding and lower values during childhood (Gardner 2013; Gardner et al. 2018). Adult males (*N* = 51) have statistically significantly higher collagen *δ* ^13^C and *δ* ^15^N values compared to adult females (*N* = 60) [*δ* ^13^C Mann–Whitney *U* test *U* = 2261.000, *p* < 0.001; *δ* ^15^N Mann–Whitney *U* test *U* = 2458.000, *p* < 0.001]. Similarly, adult males (*N* = 54) have statistically significantly higher bioapatite *δ* ^13^C values compared to adult females (*N* = 56) [bioapatite *δ* ^13^C Mann–Whitney *U* test *U* = 2097.000, *p* < 0.001]. This trend holds for the sex difference in collagen *δ* ^13^C and *δ* ^15^N and bioapatite *δ* ^13^C when males and females are compared within each temporal component [Middle Period: collagen *δ* ^13^C Mann–Whitney *U* test *U* = 149.000, *p* = 0.004; *δ* ^15^N Mann–Whitney *U* test *U* = 162.000, *p* < 0.001; bioapatite *δ* ^13^C Mann–Whitney *U* test *U* = 150.000, *p* = 0.051; Late Period: collagen *δ* ^13^C Mann–Whitney *U* test *U* = 1244.000, *p* = 0.001; *δ* ^15^N Mann–Whitney *U* test *U* = 1371.000, *p* < 0.001; bioapatite *δ* ^13^C Mann–Whitney *U* test *U* = 1121.000, *p* = 0.004]. These sex differences suggest that males consumed slightly more high trophic level protein resources, whereas females consumed more terrestrial C_3_ resources in their respective diets.

**FIGURE 5:**
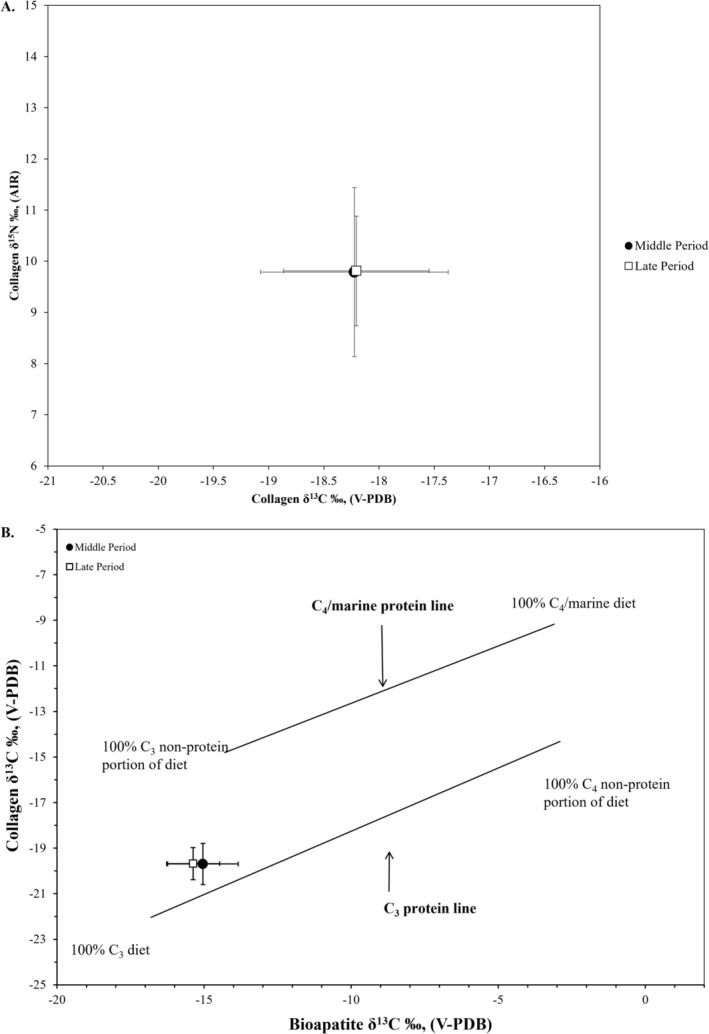
Stable isotope results plotted as sample means with 1 SD error bars for the individuals from *Mánni Muwékma Kúksú Hóowok Yatiš Túnnešte‐tka* by temporal period. The protein sources and other dietary macronutrients that impact the bone collagen *δ* ^13^C and *δ* ^15^N values show the same mean with a slightly larger standard deviation during the Middle Period (A). The Froehle et al. (2010) model indicates that when the whole diet is considered, there is no distinct patterning by temporal period, suggesting that there was not a shift in diet through time (B).

Table 2 reports the descriptive statistics for the two temporal site components, the Middle Period and Late Period. Figure 5 and the mean values reported in Table 2 show that the Middle and Late Periods are identical or nearly identical for the three isotope systems. No statistically significant differences were found between the Middle and Late Period temporal components for the entire sample, regardless of age or sex category, with one exception. For the adult females, there is a statistically significant difference in the *δ* ^15^N values between the Middle and Late Period [*δ* ^15^N Mann–Whitney *U* test *U* = 207.000, *p* = 0.044]. The female *δ* ^15^N mean values increase from 9.0‰ in the Middle Period to 9.3‰ in the Late Period, indicating a slight shift to more high trophic level protein sources consumed in their diet. However, a 0.3‰ difference in mean values is close to the instrument precision measure of ±0.2‰, so this temporal trend should be interpreted with caution. Overall, no meaningful temporal changes in diet were identified between site components at *Mánni Muwékma Kúksú Hóowok Yatiš Túnnešte‐tka*.

### 4.2 East San Francisco Bay Regional Comparisons

**FIGURE 6:**
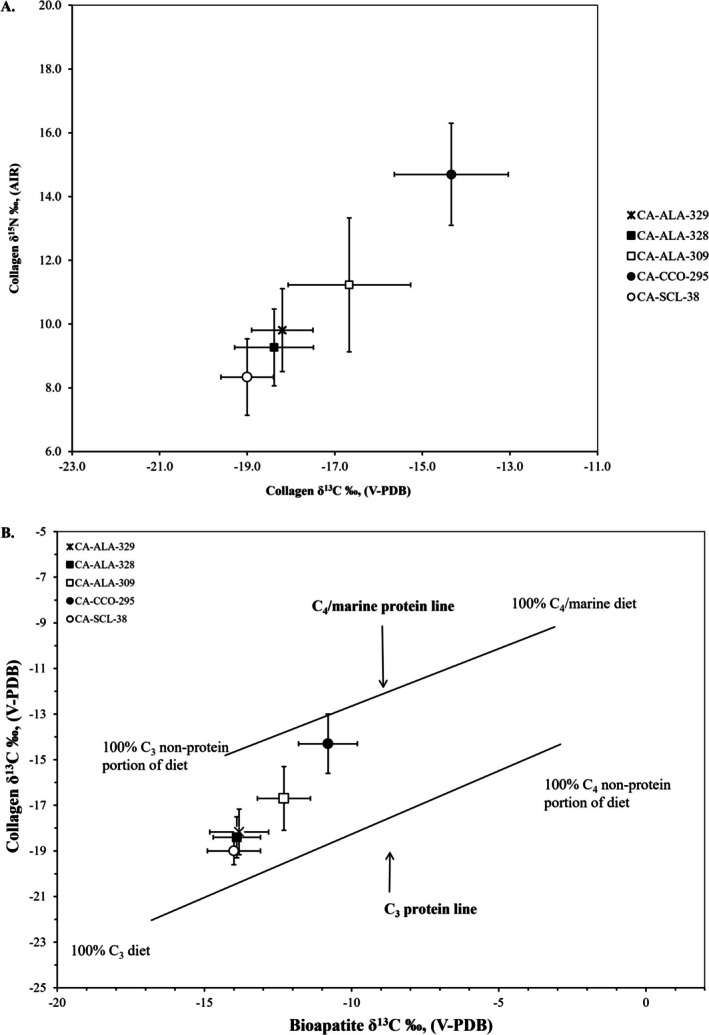
A regional comparison of stable isotope results plotted as sample means with 1 SD error bars for the adults from Middle and Late Period sites along the eastern San Francisco Bay shore. The protein sources and other dietary macronutrients that impact the bone collagen *δ* ^13^C and *δ* ^15^N values show the mean decreasing along a north to south latitude trend with some overlap in the standard deviation of the southern sites (A). The Froehle et al. (2010) model indicates that when the whole diet is considered, the same trend of decreasing values along a north to south latitude gradient is observed (B).

Given the lack of temporal change in diet at *Mánni Muwékma Kúksú Hóowok Yatiš Túnnešte‐tka*, we next compare isotope values at the site to other contemporaneous eastern bay shore sites to examine regional patterns. Table 3 presents the descriptive statistics for the five bay shore sites separated into Middle and Late Period samples, as well as with temporal components combined. To assess regional comparisons, the sample with the temporal components combined was used because it increased the sample sizes for each site and because two sites have burials assigned to a singular temporal component (Figure 6). The adults at these bay shore sites show statistically significant differences for collagen *δ* ^13^C and *δ* ^15^N values and bioapatite *δ* ^13^C values [collagen *δ* ^13^C Kruskal‐Wallis *H* (4) = 191.343, *p* < 0.001; *δ* ^15^N Kruskal‐Wallis *H* (4) = 187.455, *p* < 0.001; bioapatite *δ* ^13^C Kruskal‐Wallis *H* (4) = 154.844, *p* < 0.001]. Specifically, the collagen *δ* ^13^C values at CA‐ALA‐328 are not significantly different from *Mánni Muwékma Kúksú Hóowok Yatiš Túnnešte‐tka* or CA‐ALA‐309, but all other pairwise comparisons of the sites are significantly different from one another. The *δ* ^15^N values at CA‐CCO‐295, the most northern site on the eastern bay shore and closest to the mouth of the bay, have the highest mean value, which is significantly different from the other four sites. CA‐SCL‐38, the most southern and inland shore site, has the lowest mean *δ* ^15^N value and is also significantly different from CA‐ALA‐309 and *Mánni Muwékma Kúksú Hóowok Yatiš Túnnešte‐tka*. The bioapatite *δ* ^13^C values for the two northern sites, CA‐CCO‐295 and CA‐ALA‐309, have significantly higher mean values than the other three sites but are not different from one another. Finally, bioapatite *δ* ^13^C values for the three southern shore sites, *Mánni Muwékma Kúksú Hóowok Yatiš Túnnešte‐tka*, CA‐ALA‐328, and *Yukisma* Mound CA‐SCL‐38, are not significantly different from one another.

**TABLE 3:** Comparison of stable isotope values of adults (18+ years) by temporal component from sites along the eastern San Francisco Bay shore.

	Middle period			Late period			Combined*		
	δ^13^C (col, VPDB, ‰)	δ^15^N (col, AIR, ‰)	δ^13^C (bioapat, VPDB, ‰)	δ^13^C (col, VPDB, ‰)	δ^15^N (col, AIR, ‰)	δ^13^C (bioapat, VPDB, ‰)	δ^13^C (col, VPDB, ‰)	δ^15^N (col, AIR, ‰)	δ^13^C (bioapat, VPDB, ‰)
CA‐CCO‐295^a^ mean	−14.3	14.8	−10.8	−14.3	14.6	−10.9	−14.3	14.7	−10.8
SD	1.5	1.0	1.9	1.0	1.2	1.0	1.3	1.0	1.6
*N*	41	41	41	24	24	24	65	65	65
CA‐ALA‐309^b^ mean	−17.0	10.6	−12.5	−15.5	13.2	−11.6	−16.7	11.2	−12.3
SD	1.3	2.1	0.8	1.2	0.7	1.0	1.4	2.1	0.9
*N*	10	10	10	3	3	3	13	13	13
CA‐ALA‐329^c^ mean	−18.2	9.8	−13.7	−18.2	9.8	−13.9	−18.2	9.8	−13.8
SD	0.8	1.4	1.3	0.7	1.2	0.8	0.7	1.3	1.0
*N*	29	29	31	86	86	83	115	115	114
CA‐ALA‐328^b^ mean	−18.5	9.2	−14.0				−18.4	9.3	−13.9
SD	1.0	1.4	0.9				0.9	1.2	0.8
*N*	6	6	6				8	8	8
CA‐SCL‐38^d^ mean				−18.9	8.4	−14.0	−19.0	8.3	−14.0
SD				0.7	0.9	1.4	0.6	1.2	0.9
*N*				53	53	53	100	100	100

* Combined samples include burials at the sites that are not placed within a specific temporal component.

^a^ Beasley et al. 2013.

^b^ Bartelink 2006.

^c^ This study and Bartelink 2006.

^d^ Gardner 2013.

## 5 Discussion

In the past 20 years, extensive stable isotope research has been conducted throughout Central California, including sites along the coast, the San Francisco Bay estuary, and the Sacramento–San Joaquin Valley and Delta region. Our current study reports isotope data for 146 individuals (115 adults and 31 juveniles) from the Indigenous Ohlone site, *Mánni Muwékma Kúksú Hóowok Yatiš Túnnešte‐tka*. In keeping with the previous dietary patterns for Middle and Late Period sites along the eastern shore of the San Francisco Bay, individuals from the site consumed a mix of both marine foods and terrestrial C_3_ resources, but in different relative amounts (Figures 2 and 3). For adults, sex differences were found for both time periods—males consumed slightly more high trophic level protein resources than females, whereas females consumed slightly more terrestrial C_3_ resources (mean difference *δ* ^13^C_col_ = 0.6, *δ* ^15^N_col_ = 1.1, and *δ* ^13^C_apa_ = 0.7). Although small sex differences were found, males and females overall have similar collagen *δ* ^13^C and *δ* ^15^N values and bioapatite *δ* ^13^C values, suggesting that food resources were widely shared within households and the community (Figure 4).

No temporal changes in diet were identified between the Middle and Late Periods, even when age and sex were considered (Figure 5). Notably, the lack of temporal change in diet at the site is interesting as use of the site spans the Medieval Climatic Anomaly (MCA), a time that produced droughts across California from about ad 650 to 850 and about ad 1150 to 1250 or later (Lightfoot and Luby 2002). At this time, the three other Coyote Hills sites were abandoned, while *Mánni Muwékma Kúksú Hóowok Yatiš Túnnešte‐tka* was continually used as a burial mound through the MCA (Leventhal 1993; Lightfoot and Luby 2002). Along the bay shore, many hypotheses have been posited about the abandonment and reuse of shell mounds, such as environmental degradation, resource depression, subsistence change, population movements, and reorganization of Bay Area communities (see the detailed discussion in Lightfoot and Luby 2002). During the MCA, the southern San Francisco Bay Area sites do not have temporal evidence of behavioral changes such as increased settlement disruption, violence, malnutrition, and intensification of resources as has been documented in the greater southern California Bight (D'Oro 2009; Bartelink et al. 2019). As there is no temporal trend in dietary change at *Mánni Muwékma Kúksú Hóowok Yatiš Túnnešte‐tka*, individuals either continued to consume isotopically similar foods despite the impact that the MCA had on some food resources, or alternatively, local microhabitats were buffered from the drought by its proximity to the bay shore. The lack of temporal changes in diet during the Middle and Late Periods at the site is consistent with other eastern bay shore sites, like Ellis Landing (Beasley et al. 2013) and the Yukisma Mound (Gardner 2013), where no temporal trend was identified for those time periods. This contrasts with the more significant shift from a marine emphasis to greater consumption of terrestrial resources between the Early and Middle Periods (Bartelink 2009).

Previous research on foraging efficiency and resource intensification in the region highlights the importance of microhabitats within the landscape when considering temporal trends in faunal exploitation (Broughton 1994, 1997, 1999, 2002; Simons 1992). For example, Broughton (1994:386) used the artiodactyl index [Σ Artiodactyls_*i*_/Σ (Artiodactyls_*i*_ + Otters_*i*_)] as a measure of foraging efficiency on a regional scale and found a temporal trend that “was negative, but unimpressive”. However, when each of the subregions was analyzed individually and the sample was separated into discrete site components, significant negative relationships were found, suggesting a temporal decline in foraging efficiency (i.e., a temporal decline in artiodactyl remains and an increase in otter remains). These findings reveal that temporal patterns in faunal exploitation should be examined within the local microhabitat around the site location, rather than examining patterns using aggregated data compiled from multiple sites within a region (Broughton 1994). Of note, the increased exploitation of otters coincides with the movement of outsiders to the area, known as the *Meganos Aspect*, which likely marked a period of sociocultural transformation (Milliken et al. 2007). Hylkema (1991) has argued that the increase in otter exploitation may reflect their use for pelts more than as a food resource, given that these were prestige items and many sea otter remains lack evidence of cut marks related to food processing.

Faunal studies and isotope analysis provide different but complementary lines of evidence to understand dietary trends. Faunal remains are subject to taphonomic processes impacting the recovery of identifiable portions and can sometimes reflect the acquisition of prestige food items that may have been relatively unimportant to the overall diet. In contrast, stable isotope data are often too coarse to provide trends between different faunal species that are isotopically similar but that represent vastly different‐sized prey items (e.g., deer vs. rabbits). The significance of local microhabitats identified from faunal studies indicates that isotopic data at the local level should also be considered. To examine local microhabitats, we contextualize the *Mánni Muwékma Kúksú Hóowok Yatiš Túnnešte‐tka* individuals relative to the other four contemporaneous eastern San Francisco Bay Area sites because differences in relative proportions of marine food consumption appear to be most linked to ecogeographical partitioning of food resources along the bay shore. Today, these sites are located within a relatively small region, with the most northern site (CA‐CCO‐295) in Richmond and the most southern site (CA‐SCL‐38) in Milpitas being separated by only approximately 80.5 km (50 miles). The approximate distance from CA‐CCO‐295 to CA‐ALA‐309 is 16 km (10 miles) with the two Coyote Hills sites (CA‐ALA‐328; CA‐ALA‐329: *Mánni Muwékma Kúksú Hóowok Yatiš Túnnešte‐tka*) being 48 km (30 miles) further south along the shoreline. The most southern site (CA‐SCL‐38) is slightly inland, 24 km (15 miles) southeast of the Coyote Hills sites. The overall regional pattern occurs along a latitudinal gradient, with the highest mean *δ* ^13^C and *δ* ^15^N values in the north and the lowest mean *δ* ^13^C and *δ* ^15^N values in the south (Figure 6).

While the contribution of terrestrial C_3_ foods varied, all individuals living along the eastern bay shore consumed some marine foods given that all sites plot along a strong marine–terrestrial trendline (Figure 6). Individuals from the northeastern sites, like CA‐CCO‐295 and CA‐ALA‐309, mainly consumed local high trophic level marine resources, including fish and marine mammals, due to their proximity to the mouth of the Bay. In contrast, the individuals from the Coyote Hills area sites consumed more local terrestrial game and wild plant resources from C_3_ ecosystems. Finally, individuals from the South Bay site, CA‐SCL‐38, while still on the lowest end of the marine–terrestrial trendline of eastern bay shore sites, consumed the most local, low trophic terrestrial C_3_ resources compared to the more northern sites. Following Broughton (1994), we examined temporal patterns in the diet at *Mánni Muwékma Kúksú Hóowok Yatiš Túnnešte‐tka* but failed to identify any variation through time in stable isotope values. The lack of a temporal pattern in the stable isotope data may reflect the fact that archaeofaunal patterns do not always directly track bulk dietary consumption within a population when resources of different ranks have similar isotope values.

Most notably in this study, we identified significant regional differences, suggesting the key factor driving dietary variation is site location and latitude along the bay shore. These regional differences in diet reflect the importance of resources found within a given microhabitat, access to which would have been controlled by distinct tribal groups. Resources aggregated within tribal territories were heavily defended against outsiders, although intertribal relations between groups would have been strengthened through trade relations and intermarriage (Milliken et al. 2007). However, the low level of overlap in stable isotope values between subregions suggests that the trading of food resources, namely marine protein, was likely intermittent. Ethnographic and ethnohistoric records document instances of intertribal conflict within Central California, most often related to poaching of fishing and hunting grounds, theft of resources, and trespassing (Heizer 1978; Kroeber 1925; McCorkle 1978). Evidence of violent conflict has been documented at several sites within the Bay Area, including *Mánni Muwékma Kúksú Hóowok Yatiš Túnnešte‐tka* and the *Yukisma* Mound (CA‐SCL‐38) (e.g., R. D. Jurmain 1991; R. Jurmain 2001; Jurmain et al. 2009; Jurmain and Bellifemine 1997; Bartelink et al. 2013, 2019; Eerkens et al. 2014). Allen et al. (2016) attribute the high prevalence of projectile trauma and other types of sharp‐force injuries in human burials from this region to resource scarcity, suggesting a relationship between high levels of violence and low resource productivity within a region. Further, they found no clear patterns between levels of violence and political organization or political leadership. Thus, this argument suggests that the motivation to defend resources is more closely tied to ecogeographic partitioning on the landscape.

If different tribal groups, represented by different isotopic values at sites along a latitudinal gradient, defended access to a given microhabitat with only intermittent trade of marine foods, then dietary isotope values might provide insight into the movement of individuals between tribes. Traditionally, isotope methods used for understanding movement on the landscape, whether region‐of‐origin or migration, focus on the use of strontium, lead, oxygen, and hydrogen isotopes. However, in modern forensic applications, dietary isotopes, specifically carbon, have been shown to provide geolocation information (Bartelink et al. 2014). In that same vein, in densely populated regions where competition over food resources might have resulted in distinct boundaries that dictated access to food resource patches defended by tribal groups, individuals with a non‐local dietary signature might have spent significant time as an adult consuming resources from a different ecogeographic microhabitat.

For example, at *Mánni Muwékma Kúksú Hóowok Yatiš Túnnešte‐tka*, four adults (male burials 61 and 120; female burials 51 and 158) have collagen *δ* ^13^C and *δ* ^15^N values that are more than two standard deviations below the mean values for males and females from the site. The collagen dietary isotope values are more similar to individuals from CA‐SCL‐38 to the south who have the lowest values along the eastern bay shore. The four individuals fall within two standard deviations of the mean values for CA‐SCL‐38 except Burial 51, a female, who has the lowest *δ* ^15^N value (5.4‰), also lower than two standard deviations for the CA‐SCL‐38 female mean value. However, all four individuals have bioapatite *δ* ^13^C values within the two standard deviations from the mean for the *Mánni Muwékma Kúksú Hóowok Yatiš Túnnešte‐tka* site. The opposite is true for seven individuals (male burials 169, 243, and 249; female burials 76, 113,153, and 278) who have collagen *δ* ^13^C and *δ* ^15^N values within two standard deviations but higher bioapatite *δ* ^13^C values that are more than two standard deviations above the mean values for males and females from the site. The seven bioapatite *δ* ^13^C values are within one standard deviation of the combined adult mean values for CA‐CCO‐295 and CA‐ALA‐309. As none of the individuals have both collagen and bioapatite values that are outliers, these differences may represent food preferences rather than the movement of people along the bay shore. However, it is interesting that all individuals with collagen outlier values look similar to the most southern site (CA‐SCL‐38), while all bioapatite individual outliers look more similar to the northern sites (CA‐ALA‐309 and CA‐CCO‐295). There is no temporal pattern or relationship to the MCA in assumed movement between sites based on the isotope values as both males and females move both to the north and south across all temporal phases.

## 6 Conclusion

In the densely populated San Francisco Bay Area, foraging efficiency was localized, suggesting that the exploitation of different microhabitats was critical to each tribal group along the bay shore. Populations closest to the mouth of the Bay consumed more high trophic level protein resources (e.g., marine and anadromous fish, marine mammals, waterfowl) and incorporated fewer terrestrial C_3_ resources (e.g., terrestrial game, acorns, small seeds) compared to their more southern neighbors. Any trade of marine resources between neighboring groups along the eastern bay shore did not likely occur with enough regularity to homogenize the isotope values of individuals from different sites as seen in other regions in Central California, such as sites in the Sacramento‐San Joaquin Valley and Delta region (Bartelink 2009). Except for the two Coyote Hill sites (*Mánni Muwékma Kúksú Hóowok Yatiš Túnnešte‐tka* and CA‐ALA‐328), which are isotopically similar, sites within the four ecogeographical regions have isotopically distinct diets that all fall along a trendline gradient of varied consumption of marine versus C_3_‐based terrestrial resources. This suggests that individuals from each site consumed isotopically distinct food resources, incorporating different relative amounts of marine and C_3_ terrestrial resources. Stable isotope values can highlight trends in the diet of past populations to test models of resource intensification and ecogeographic partitioning of microhabitats. But populations are composed of individuals. An individual's osteobiography from dietary isotopes should also be considered when interpreting isotope data to understand life history patterns such as movement on the landscape, interactions with different ecogeographic zones with defined food resources, and dietary preferences within the larger context of a population. Ongoing collaboration with the Muwekma Ohlone Tribe will continue to explore the relationship between diet, burial context, and the mortuary assemblages at *Mánni Muwékma Kúksú Hóowok Yatiš Túnnešte‐tka*.

## Author Contributions

**Melanie M. Beasley:** conceptualization (equal), data curation (lead), formal analysis (lead), funding acquisition (lead), methodology (lead), resources (equal), writing – original draft (equal). **Eric J. Bartelink:** conceptualization (equal), data curation (supporting), formal analysis (supporting), funding acquisition (supporting), methodology (supporting), writing – original draft (equal). **Alan Leventhal:** resources (equal), writing – review and editing (equal). **Monica V. Arellano:** writing – review and editing (supporting). **Richard Massiatt:** writing – review and editing (supporting). **Charlene Nijmeh:** writing – review and editing (supporting).

## Supporting information


Data S1.

Data S2.

## Data Availability

The raw data for the study of the Mánni Muwékma Kúksú Hóowok Yatiš Túnnešte‐tka population are available in the supplementary information of this article.
